# Kinesiophobia in patients with heart failure: concept analysis using Rodgers' evolutionary approach

**DOI:** 10.3389/fcvm.2025.1703928

**Published:** 2025-12-11

**Authors:** Zhuzhu Qin, Huanju Liu, Yining Tao, Yan Shen, Yuxuan Wu, Qin Huang, Xiaoling Zou, Yukun Zhang, Xinxin Ye

**Affiliations:** 1 Department of Nursing, Peoples Hospital of Deyang City, Deyang, Sichuan, China; 2 Department of Medical Genetics, Naval Medical University, Shanghai, China; 3 Clinical Research Center, Ningbo No. 2 Hospital, Ningbo, Zhejiang, China; 4 Operating Room, People’s Hospital of Zhongjiang, Deyang, Sichuan, China; 5 Department of Nursing, the Second Affiliated Hospital of Zhejiang University School of Medicine, Hangzhou, China; 6 Department of Sports Science, Zhejiang University, Hangzhou, Zhejiang, China

**Keywords:** concept analysis, kinesiophobia, heart failure, Rodgers' approach, cardiac rehabilitation

## Abstract

**Background:**

Kinesiophobia, a prevalent and multifaceted issue among patients with heart failure (HF), significantly impedes physical activity, hampers disease management, and delays recovery. An analysis of kinesiophobia can clarify its characteristics and inform strategies for improving patient care and rehabilitation.

**Objective:**

To establish a comprehensive conceptual model of kinesiophobia in patients with HF by systematically analyzing its attributes, antecedents, and consequences using Rodgers' evolutionary method.

**Method:**

A concept analysis using Rodgers' evolutionary method identified the attributes, antecedents, and consequences of kinesiophobia. A systematic search of Web of Science, Embase, PubMed, CINAHL, and PsycInfo yielded 30 articles published from database inception to 2025 for analysis.

**Results:**

Kinesiophobia is characterized by four attributes: symptom distress, complex emotional responses, avoidance behavior, and cognitive bias. Its antecedents include demographic characteristics, disease-related factors, psychological and emotional factors, physical functions and coping strategies, and social support status. The consequences of kinesiophobia encompass three main themes: physical deterioration and increased health risks, psychological burden, and impaired disease management and recovery.

**Conclusion:**

This concept analysis enhances understanding of kinesiophobia in HF patients, offering insights into the factors influencing fear of movement and emphasizing the need for early identification and targeted interventions. This understanding can guide clinicians in promoting safe physical activity, improving rehabilitation adherence, and enhancing patient recovery and well-being.

## Introduction

Heart failure (HF) constitutes a major global public health challenge, characterized by high prevalence, escalating healthcare costs, and significant impairment in patients' quality of life. As a chronic and progressive syndrome, HF often leads to frequent hospitalizations, functional decline, and increased mortality (1). Current estimates indicate that more than 64 million people worldwide are living with HF, placing an substantial burden on healthcare systems (2). Given the poor long-term prognosis associated with HF, identifying effective strategies to enhance functional capacity and quality of life has become an urgent priority. Exercise training, a cornerstone of multidisciplinary cardiac rehabilitation, is a Class IA recommendation in international guidelines for the management of HF, proven to improve exercise capacity, reduce symptoms of dyspnea and fatigue, enhance quality of life, and lower the risk of hospital readmission (3–5). However, a striking gap persists between the proven clinical efficacy of exercise interventions and their limited implementation in real-world practice.

Despite robust evidence supporting its benefits, adherence to prescribed physical activity and engagement in exercise-based rehabilitation programs remains critically low among the HF population (6, 7). Among the diverse barriers to participation, psychological factors have been increasingly recognized as pivotal obstacles. A pivotal psychological barrier that negatively impacts engagement in physical activity is kinesiophobia, an excessive and irrational fear of movement and physical activity rooted in belief that exercise may trigger adverse cardiac events or worsen symptoms (8, 9). Emerging research suggests that kinesiophobia is highly prevalent in HF. A systematic review and meta-analysis revealed its prevalence to be as high as 69.2% among this patient population (10). This psychological barrier is independently associated with reduced physical function, lower activity levels, poorer health-related quality of life, and increased mortality (11–13).

While the construct of kinesiophobia originated in the context of chronic musculoskeletal pain, where it is defined as a fear of movement due to pain re-injury beliefs (14, 15), its manifestation in HF is qualitatively distinct, reflecting a unique clinical context. The fear in HF patients is not primarily of pain, but of triggering dyspnoea, palpitations, extreme fatigue, or acute cardiac decompensation, which are perceived as life-threatening (12). This fear is often reinforced by the unpredictable and episodic nature of HF symptoms, leading to maladaptive avoidance behaviours and a vicious cycle of deconditioning and worsening symptom burden (16). Although the concept has been increasingly studied in cardiovascular populations, its application in HF remains nascent and conceptually ambiguous. The term is often used interchangeably with related constructs such as fear of movement or activity avoidance, leading to inconsistent operationalization, measurement challenges, and a lack of targeted interventions (17, 18). Taken together, kinesiophobia in HF remains an evolving and poorly delineated construct, highlighting the need for systematic clarification of its attributes and boundaries.

Concept analysis serves as a rigorous methodological approach to clarify the structure, attributes, and dynamics of complex phenomena. Rodgers' evolutionary method of concept analysis is particularly suited to this endeavour, as it acknowledges that concepts are not static but evolve over time and are shaped by their contextual use (19). This method facilitates a systematic examination of the antecedents, attributes, consequences, surrogate terms, and related concepts of kinesiophobia within the unique context of HF. By tracing its evolutionary pathway and synthesizing its current use across disciplines, this approach provides a solid foundation for theoretical development and practical application.

This study aims to conduct a systematic concept analysis of kinesiophobia in HF patients using Rodgers' evolutionary framework. We hypothesize that kinesiophobia in HF is a multidimensional construct influenced by cognitive-perceptual, emotional, and social factors, which evolves throughout the disease trajectory. The analysis will elucidate the core attributes of the concept, differentiate it from related terms, and present a clear, evidence-based definition. The findings are expected to provide a refined conceptual model to guide future research, improve clinical assessment, and inform the development of tailored psychological and rehabilitative strategies to address kinesiophobia in this vulnerable population.

## Methods

The present study employed Rodgers'evolutionary method to conduct a concept analysis of kinesiophobia in patients with HF (19). This inductive methodology was selected to elucidate the conceptual structure of this phenomenon by examining how it is defined, characterized, and utilized across a breadth of literature. The approach is grounded in the understanding that concepts are dynamic and context-dependent, continually shaped by clinical practice and patient experience. Given that the conceptualization of kinesiophobia in heart failure is evolving, this method provides the ideal framework to rigorously identify its core attributes, antecedents, and consequences, thereby establishing a comprehensive and contextually-specific understanding.

### Sample and data collection

#### Inclusion and exclusion criteria

**Inclusion criteria:** Studies involving patients with HF that focused on the concept of kinesiophobia, including its defining attributes, antecedents, consequences, influencing factors, and related outcomes.

**Exclusion criteria**: Duplicate publications, articles with inaccessible full texts, studies unrelated to the research topic, and non-English literature. Two researchers independently screened the literature, extracted data, and categorized the findings. Any disagreements were resolved through discussion or by consulting a third researcher to reach a consensus.

#### Search strategy

In this study, the English search terms used for the retrieval were “heart failure/ cardiac failure/ heart insufficiency/ heart decompensation/ congestive heart failure/ kinesiophobia/ fear of movement/ pain related activity avoidance/ movement phobia/ movement fear/ fear of exercise”, targeting the following databases: PubMed, Web of Science, Embase, CINAHL, and PsycInfo. The retrieval strategy was tailored to the indexing and search characteristics of each database, and the search period extended from the inception of each database up to August 16, 2025.

### Data analysis

Rodgers' evolutionary method was employed to inductively explore and clarify the concept of kinesiophobia in HF patients (19). This approach guided a structured process consisting of six steps: (1) identifying the concept of interest, surrogate terms, and relevant uses in the literature; (2) selecting appropriate sources of evidence from cardiology, rehabilitation, psychology, and nursing databases; (3) collecting and organizing data to reveal patterns of use and defining features; (4) conducting thematic analysis to specify attributes, antecedents, and consequences; (5) identifying related or overlapping concepts, such as exercise intolerance, anxiety, or avoidance behavior; and (6) recognizing theoretical and practical implications that could inform research and clinical assessment.

To systematically manage the data, a standardized data extraction matrix was developed, which is presented as Table 1. Two researchers independently reviewed the full text of all 30 included articles. They extracted verbatim phrases, keywords, and concepts related to the attributes, antecedents, and consequences of kinesiophobia in patients with HF. The extracted data were then organized into the three corresponding columns in Table 1 for each study. This table served as the foundational dataset for the subsequent thematic analysis, allowing for a clear overview of the conceptual components across the literature before synthesis. Finally, findings were reviewed and refined through expert consensus discussions, ensuring conceptual clarity, clinical relevance, and applicability to future rehabilitation strategies.

**Table 1:** Characteristics and conceptual data extraction of included studies (*n* = 30).

Author (Year)	Sample size	Man, *n* (%)	Age, years (mean ± SD)	Type of HF	NYHA class	Attributes	Antecedents	Consequences
Zhang et al., (40)	305	185 (60.7)	64.6 ± 10.7	NA	II/III	Perceived control	Fatigue, Exercise self-efficacy	Quality of life
Westas and Melnikov, (31)	Review NA	NA	NA	NA	NA	Emotional reactions, Subjective avoidance behavior	Anxiety, Depression, Sleep disorders, Multiple comorbidities, Subjective social status	Disease management
Tian et al., (35)	248	133 (53.6)	72.04 ± 0.46	NA	II/III/IV	Cognitive bias	Social support, Illness perception, Resilience	Disease management
Lüke et al., (36)	82	61 (74.4)	61.0 ± 11.0	(1) 51 (62.2); (2) 17 (20.7); (3) 13 (15.9)	I/II/III	Cognitive avoidance	Coping dispositions	
Cheng, (16)	300	158 (52.7)	60.84 ± 6.32	CHF	I/II	Perceived control	Heart failure symptoms, Family functioning status, Social support	
Zeng et al., (32)	Review NA	NA	NA	NA	NA	Emotional response, Subjective avoidance Behavior, Misperception	Age, Gender, Educational level, Economic level, Pain severity, Frailty, Exercise self-efficacy, Disease-related factors, Psychosocial factors	Reduced recovery, Prolonged hospital stays, Decreased quality of life
Xiang et al., (37)	NA	NA	NA	NA	NA	Emotional response, Subjective avoidance Behavior, Disease awareness	Socio-demographic factors, Psychological, Cognitive factors, Disease Treatment factors, Lifestyle	Increase psychological pressure, Reduce treatment compliance
Wang et al., (38)	NA	NA	NA	NA	NA	Emotional response, Subjective avoidance behavior	Family support level, Exercise self-efficacy, Fatigue, Age, Education level, Disease course, Coexisting diseases, Family support level, Fatigue.	Reduce treatment compliance
Spaderna et al., (18)	185	140 (75.7)	61.6 ± 11.4	(a) 114 (61.6) (2) 43 (23.2) (3) 26 (14.1)	I/II/III/IV	Hypervigilance, Fear of cardiac events	Attention towards and being distressed by HF symptoms, Heart failure drugs, Antidepressants, Coping styles	Health threats
Sentandreu-Mañó et al., (8)	107	61 (57)	73.18 ± 12.68	NA	NA	Fear of cardiac events, Subjective avoidance behavior	Musculoskeletal pain, Quality of sleep, Functional capacity, Disability, Being at risk of frailty	Participation and adherence, Quality of life
Liu et al., (10)	NA	NA	NA	NA	NA	Fear of cardiac events	Education level, Monthly income, Anxiety, Exercise self-efficacy	participation and adherence
Li et al., (46)	NA	NA	NA	NA	NA	Subjective avoidance behavior	Exercise self-efficacy	Decreased compliance with rehabilitation exercises
Li et al., (45)	318	200 (62.89)	68.78 ± 14.53	NA	II/III	Fear of cardiac events, Negative emotion (pain catastrophizing)	Rumination and psychological resilience, symptom burden	Increase the risk of cardiovascular damage
Keessen et al., (44)	116	97 (83.6)	65.5 ± 5.82	NA	NA	Pain catastrophizing	Pain, Physical activity intensity	Low physical activity levels, Long disease duration, Decreased muscle strength, Reduced quality of life
Enzin et al., (39)	217	105 (48.4)	61.03 ± 11.99	NA	NA	Subjective avoidance behavior	Age, Physical adaptation, Psychological adaptation	
Zhang et al. (13)	305	185 (60.7)	64.41 ± 8.38	NA	II/III	Multiple emotional responses	Cardiac anxiety, Depressive symptoms, Subjective social status, Employment status	
Yifan et al., (9)	244	138 (56.6)	63.1 ± 12.0	CHF	I/II/III/IV	Concerns about symptoms	Activities of daily living, Heart function	Quality of life
Sentandreu-Mañó et al., (15)	107	61 (57.0)	73.18 ± 12.68	NA	NA	Pain catastrophizing	Musculoskeletal pain, Age	Quality of life
Ratnoo et al., 2023	300	258 (86)	59.1 ± 8.6	NA	NA	Subjective avoidance behavior	Mental condition	Self-reported disability
Qin et al., (11)	270	131 (48.5)	76.17 ± 7.96	CHF	II/III	Emotional response, Subjective avoidance behavior	Symptoms of HF, Coping mode, Exercise self-efficacy, Social support	Physical health, Quality of life
Sempere-Rubio et al., 2022	100	58 (58.0)	71.20 ± 13.19	NA	NA	Pain catastrophizing, Subjective avoidance behavior	Activities of daily living	Quality of life
Qin et al., (26)	236	120 (50.8)	67.1 ± 9.2	NA	II/III	Concerns about symptoms	Educational background, Monthly family income, Disease course, Fatigue	Physical health
Keessen et al., (34)	149	116 (77.9)	64.1 ± 10.1	NA	NA	Emotional response, Subjective avoidance behavior	Age, Anxiety, Psychological complexity	Low physical activity levels, Reduced quality of life
Keessen et al., (28)	16	10 (62.5)	64.5 ± 11.55	NA	NA	Negative beliefs and attitudes	Reduced physical activity	Non-adherence to cardiac rehabilitation
Hoffmann et al., (33)	60	39 (65.0)	66.0 ± 7.6	NA	NA	Subjective avoidance behavior	Defensive responses on a physiological level	Low physical activity levels
Hoffmann et al., (17)	132	106 (80.3)	67.1 ± 12.1	NA	II/III/IV		Exercise intensity, Depression, Complications	Physical health, Quality of life
Gołba et al., (41)	101	73 (72.3)	61.9 ± 13.56	NA	IV		Age, Heart function, Hemoglobin level	Physical health, Quality of life
Ha et al., (25)	NA	NA	NA	NA	NA	Painful experience	Exercise self-efficacy	Reduce treatment compliance
Brunetti et al., (43)	58	39 (66)	59.5 ± 11.9	AHF	NA	Beliefs, Emotional response	Age, Educational background	Quality of life
McKelvie et al., (42)	NA	NA	NA	NA	NA		Peripheral changes in skeletal muscle and blood supply	Reduce treatment compliance

SD, standard deviation; NA, not applicable; CHF, chronic heart failure; (1) HFrEF, heart failure with reduced ejection fraction; (2) HFmrEF, heart failure with mildly reduced ejection fraction; (3) HFpEF, heart failure with preserved ejection fraction; NYHA, New York Heart Association; AHF, acute heart failure; Attributes refer to the defining characteristics or features of kinesiophobia. Antecedents are the events or factors that precede the occurrence of kinesiophobia. Consequences are the outcomes or results that follow from kinesiophobia. An empty cell indicates that the study did not explicitly provide information for that specific conceptual component.

## Results

### Characteristics of the study populations

**Figure 1:**
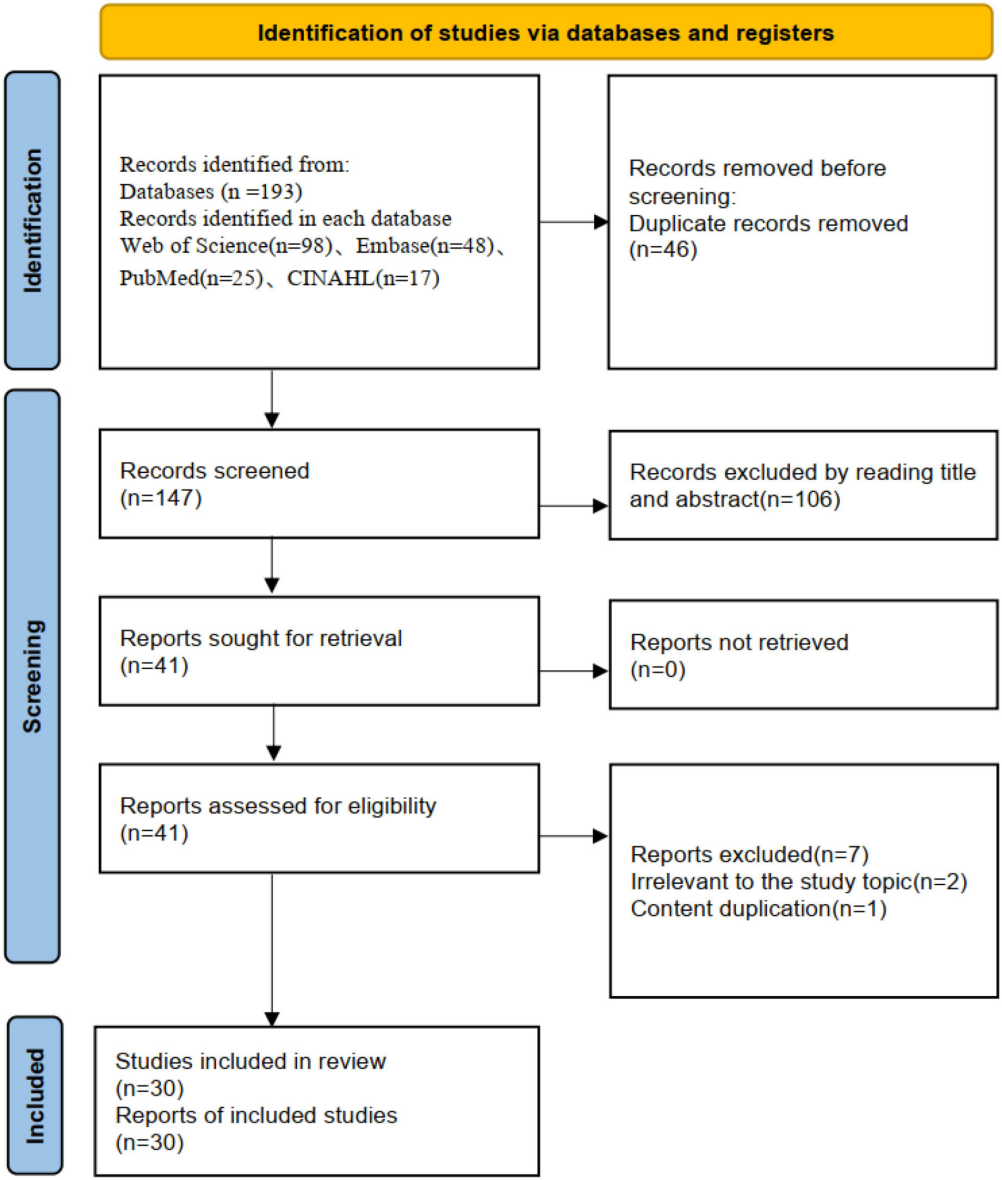
Flowchart of the study selection process of the concept analysis.

The study selection process is illustrated in the PRISMA 2020 flow diagram in Figure 1 (20), and the detailed data extraction table for the included studies is presented in Table 1. In general, the participants were older adults, typically in their 60s and 70s, with a predominance of male patients. The studies primarily involved individuals with symptomatic chronic heart failure, most commonly classified within New York Heart Association (NYHA) Class II and III. While a broad spectrum of patients was included, the specific type of heart failure (e.g., HFrEF, HFpEF) was often not reported.

### Evolution of the concept

The concept of kinesiophobia originated from chronic pain research and is grounded in the fear-avoidance model proposed by Lethem et al. (21). In 2008, de Souza et al. (22), introduced the term to describe heightened fear of physical activity stemming from pain sensitivity. The related term, fear of physical activity (FoPA), encompasses not only fear of movement but also mistrust in one's physical capacity, concerns about injury or failure, and social anxiety. The extension of kinesiophobia to cardiovascular populations began in the 2010s. It was first applied to patients with coronary heart disease to explain non-participation in cardiac rehabilitation (23) and was subsequently adapted for the HF population (24). In this new context, the conceptual focus broadened beyond pain to encompass a multidimensional fear related to cardiac symptoms. Subsequent studies in HF further specified these dimensions, linking kinesiophobia to cognitive misinterpretations of physiological responses (25), fatigue (26), and musculoskeletal pain (15).

#### Surrogate terms of kinesiophobia in patients with heart failure

In the context of HF patients, several terms have been used interchangeably with kinesiophobia to describe patients' fear or avoidance of physical activity. These include fear of movement, FoPA, exercise-related fear, and movement avoidance (27, 28). Fear of movement and FoPA are the most widely used, emphasizing patients' anxiety about engaging in activity due to concerns about worsening symptoms, experiencing fatigue, or provoking adverse cardiac events. Movement avoidance highlights the behavioral outcome of this fear, reflecting the tendency to reduce or avoid exercise, while exercise-related fear captures the emotional and anticipatory aspects of perceived exercise risk in HF patients.

#### Related concepts of kinesiophobia in patients with heart failure

Several concepts are closely related to kinesiophobia in HF patients, reflecting cognitive, behavioral, and psychosocial dimensions. Exercise self-efficacy refers to patients' confidence in their ability to safely perform physical activity, influencing participation and adherence to exercise programs (25). Health-related anxiety reflects heightened concern about symptom exacerbation or adverse outcomes during physical activity (29). Fatigue perception and musculoskeletal pain are physiological factors that contribute to avoidance behavior, mediating the relationship between fear and reduced activity (8, 15). While surrogate terms describe the direct fear or avoidance of movement, these related concepts provide a broader framework explaining why HF patients may limit exercise, integrating cognitive appraisal, emotional response, and physical limitations. Together, these terms and concepts define the multidimensional nature of kinesiophobia in HF.

### Synthesis of conceptual components

The conceptual components of kinesiophobia—its attributes, antecedents, and consequences—were inductively derived from the literature detailed in Table 1. This analytical process involved an iterative comparison, interpretation, and conceptual grouping of the various terms and descriptions reported across the 30 included articles, allowing them to be organized into coherent conceptual categories. This systematic processing of the data formed the foundation for the subsequent development of the conceptual model of kinesiophobia in HF, which is elaborated in the following sections.

### Attributes of kinesiophobia in patients with heart failure

#### Symptom distress

Symptom distress is a key attribute of kinesiophobia in HF patients, referring to the heightened awareness and emotional reaction to physical sensations such as fatigue, shortness of breath, and palpitations (30). In clinical research, this subjective burden is operationalized in various ways; it is sometimes quantified as “symptom status” via instruments like the Symptom Status Questionnaire-Heart Failure (SSQ-HF) (11), or otherwise richly described through qualitative analyses of patient experiences. It includes the misinterpretation of normal physiological responses as signs of disease exacerbation and fear of acute events.

#### Complex emotional responses

Complex emotional responses refer to the range of negative emotions, including fear, anxiety, and helplessness, that HF patients experience in response to the threat of physical activity. These emotions often stem from the patient's beliefs about the disease and its progression. In the synthesized clinical research, these emotional states are quantified using validated instruments. For example, anxiety is commonly assessed with the Generalized Anxiety Disorder-7 (GAD-7) scale, while depressive symptoms are measured using the Patient Health Questionnaire-9 (PHQ-9) (31, 32).

#### Avoidance behavior

Avoidance behavior is a defining characteristic of kinesiophobia in patients with HF, manifesting as the deliberate reduction of physical activity due to the fear of potential negative outcomes. In clinical research, this behavior is often quantified by inversely measuring exercise adherence, using tools such as the Home-Based Cardiac Rehabilitation Exercise Adherence Scale and Mainz Coping Inventory (12, 18). This behavior is often driven by the patient's perception that physical exertion could lead to symptom exacerbation or health deterioration (33, 34).

#### Cognitive bias

Cognitive bias in HF patients refers to the distorted ways in which they interpret physical sensations, particularly those related to exertion. In clinical research, such cognitive biases are often quantified using instruments designed to assess a patient's beliefs and understanding of their illness, such as the Brief Illness Perception Questionnaire (BIPQ) (35). After experiencing distressing events, such as dyspnea or fatigue during physical activity, patients may develop a tendency to misinterpret normal physiological responses as signs of disease progression (35, 36). This was also associated with a limited understanding of the benefits of exercise (25, 37).

### Antecedents of kinesiophobia in patients with heart failure

#### Demographic characteristics

The literature identified associations between kinesiophobia and several demographic factors (32, 37, 38). Older age is consistently reported as a key antecedent (39). This is consistent with the characteristics of the populations in the studies reviewed, where participants were predominantly older adults with mean ages typically in their 60s and 70s (Table 1). Regarding gender, some evidence suggests that female patients tend to report higher levels of kinesiophobia compared to their male counterparts. Additionally, other factors such as lower education levels, reduced income (26), and unemployment status have also been linked to increased kinesiophobia.

#### Disease-Related factors

Disease-related factors are crucial in understanding the antecedents of kinesiophobia in HF patients. The severity of HF symptoms, as quantified by the New York Heart Association (NYHA) functional classification, is a significant predictor. Studies indicate that a higher NYHA classification is associated with greater levels of kinesiophobia, even among patients with mild to moderate symptoms (i.e., ranging from NYHA Class I to III) (16, 40). Moreover, the presence of multiple comorbidities such as diabetes, hypertension, and arthritis (17); and physiological markers like low hemoglobin levels and poor cardiac function, as measured by ejection fraction (41, 42).

#### Psychological and emotional factors

Psychological and emotional factors are central to the development of kinesiophobia in HF patients. Anxiety, depression, and fear of cardiac events are frequently reported in individuals with HF and are significant contributors to their avoidance of physical activity (31, 37). Other identified factors include a history of negative experiences, such as discomfort during exercise (43), feelings of helplessness, and negative beliefs that frame exercise as too risky or beyond personal capabilities (12).

#### Physical functions and coping strategies

Physical function and coping strategies are closely linked to the development of kinesiophobia in HF patients. This includes reduced physical function, such as limited walking capacity or chronic pain, which was associated with a higher likelihood of avoiding activity (17, 44). Additionally, the use of maladaptive coping strategies, like avoidance or denial, was linked to higher levels of kinesiophobia (18, 36). Conversely, adaptive factors such as high self-efficacy and a belief in the benefits of exercise were associated with a greater willingness to participate in physical activities (25, 40).

#### Social support status

Social support is an essential factor in the development and management of kinesiophobia in HF patients. The presence of strong social encouragement and emotional support was reported to alleviate the fear of movement and build confidence in performing physical activities safely (16, 35). In contrast, the literature indicates that a lack of support from family, friends, or healthcare providers is associated with higher kinesiophobia (26, 38).

### Consequences of kinesiophobia in patients with heart failure

#### Physical deterioration and increased health risks

Avoidance of physical activity due to kinesiophobia leads to significant physical decline. This deterioration manifests primarily as a quantifiable reduction in daily physical activity. In the literature we analyzed, this decline is objectively measured using wearable devices like accelerometers or Personal Activity Monitors (PAM), which track key metrics such as lower daily step counts and reduced time spent in moderate-to-vigorous physical activity (MVPA) (36). This reduction in activity contributes to consequences like decreased muscle strength and, in advanced cases, observable muscle wasting. Over time, these functional impairments lead to broader negative outcomes, including decreased mobility, a heightened risk of disability, and a worse clinical prognosis, often marked by more frequent cardiovascular complications and hospitalizations (32, 45).

#### Psychological burden

Kinesiophobia places a substantial psychological burden on patients, leading to heightened levels of anxiety, depression, and stress. The fear of movement often stems from a lack of control over symptoms and uncertainty about the future, intensifying feelings of helplessness (37). This emotional distress not only reduces overall quality of life but also discourages participation in rehabilitation or physical activity programs, further compounding the negative physical health outcomes.

#### Impaired disease management and recovery

Kinesiophobia hampers effective disease management and slows recovery. Patients who are fearful of movement are less likely to engage in exercise regimens, which are essential for improving heart function and overall health. Reduced physical activity impairs cardiovascular rehabilitation, leading to prolonged hospital stays and slower recovery (13, 38). This lack of adherence to exercise programs not only exacerbates symptoms but also delays recovery and increases the risk of hospital readmission. Over time, this results in a diminished capacity to manage the disease, further compounding the challenges of HF management (8, 44).

## Model case

Mr. Zhang, a 65-year-old male living with his wife, was diagnosed with heart failure (HFrEF, LVEF 35%) two years ago. His condition has since progressed, and he now experiences symptoms consistent with NYHA Class III, such as severe fatigue and shortness of breath that have worsened over time. Despite his cardiologist's recommendation for cardiac rehabilitation, he developed a strong fear of physical activity, believing any exertion could provoke a fatal cardiac event. He avoids even simple tasks like walking or household chores. He has not sought psychological support and often expresses profound feelings of loss, believing he will never return to his former life. His medical history includes hypertension and diabetes, which worsen his HF symptoms, and he frequently experiences chest discomfort. Although offered opportunities for cardiac rehabilitation, he has avoided them due to his fears. Recently, his physical condition has declined, making walking difficult. He was hospitalized for a minor heart event, which increased his psychological burden. Despite advice to increase physical activity for recovery, his fear of exercise has slowed his progress.

## Discussion

**Figure 2:**
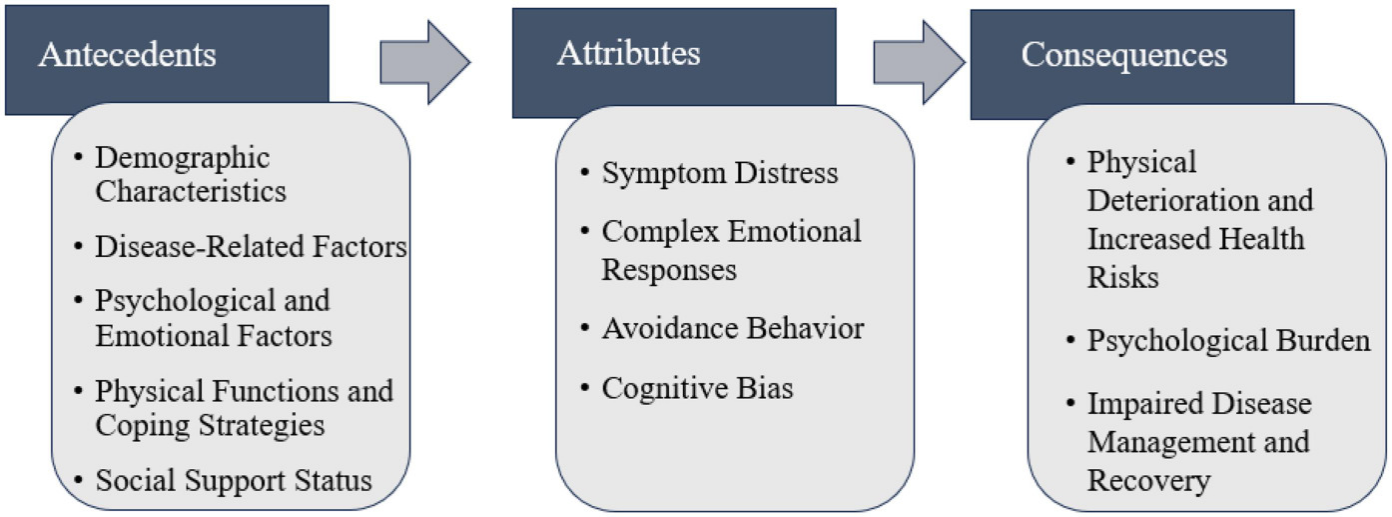
Conceptual model of kinesiophobia in patients with HF.

This concept analysis elucidates the meaning and dimensions of kinesiophobia in patients with HF using Rodgers' evolutionary approach. The findings identified four defining attributes—symptom distress, complex emotional responses, avoidance behavior, and cognitive bias—five antecedents, and three categories of consequences. Collectively, these results underscore that kinesiophobia in HF is a multidimensional phenomenon involving the interplay of physical, psychological, and social factors, ultimately hindering disease management and rehabilitation. Based on this analysis, we proposed a conceptual model of kinesiophobia in HF patients, depicted in Figure 2.

In the HF population, several surrogate terms—such as fear of movement, FoPA, exercise-related fear, and movement avoidance—have been used interchangeably with kinesiophobia (40). Although these terms describe overlapping phenomena, they differ in emphasis. Fear of movement and FoPA focus on patients' anxiety regarding potential symptom exacerbation or cardiac events during activity, whereas movement avoidance stresses the behavioral outcome of fear (36). In contrast, exercise-related fear captures the anticipatory emotional reaction before engaging in activity. Related concepts, including exercise self-efficacy, health-related anxiety, and fatigue perception, provide important context but are not synonymous (31, 40, 46). For example, low self-efficacy may predispose patients to kinesiophobia, while anxiety and fatigue contribute to its maintenance. Thus, kinesiophobia can be distinguished from related constructs by its integration of cognitive appraisal, emotional response, and avoidance behavior, reflecting a fear–avoidance cycle specific to the experience of HF.

The four defining attributes collectively depict the essence of kinesiophobia in HF. Symptom distress reflects patients' heightened sensitivity to bodily sensations, such as breathlessness or fatigue, which are often misinterpreted as signs of deterioration (26). These sensations trigger complex emotional responses, including fear, anxiety, and helplessness, which shape negative beliefs about exercise (31). Avoidance behavior subsequently emerges, as patients deliberately restrict daily activities and exercise to minimize discomfort (32, 37). Cognitive bias reinforces this cycle by promoting catastrophic interpretations of normal physiological reactions, thereby perpetuating fear and avoidance.

The antecedents of kinesiophobia are multifactorial. Demographic factors, such as older age and lower education were frequently associated with greater fear, while disease-related factors—including symptom severity and comorbidities—further heightened risk (10, 38). Psychological and emotional vulnerabilities, such as anxiety, depression, and fear of cardiac events, play a central role (13, 31). Physical limitations and maladaptive coping strategies also contribute to avoidance, while insufficient social support reduced patients' confidence in safely engaging in physical activity (38). These findings emphasize that kinesiophobia does not arise from a single determinant but rather from the convergence of physical, psychological, and social conditions.

The consequences of kinesiophobia in HF are consistently negative. Avoidance of physical activity leads to progressive physical deterioration, including muscle weakness, reduced mobility, and increased cardiovascular risk (45). Psychologically, patients experience heightened anxiety, depression, and distress, which in turn reduce quality of life (38). From a clinical perspective, impaired adherence to rehabilitation programs and delayed recovery are evident, resulting in poorer disease management and higher risk of hospital readmission. This fear–avoidance cycle consequently contributes to a downward spiral of physical and emotional decline, ultimately threatening independence and long-term prognosis.

Previous studies of kinesiophobia have largely focused on musculoskeletal conditions, where fear is directed toward pain or injury (8). In HF, however, the focus of fear shifts to cardiovascular risks, such as provoking dyspnea, chest pain, or sudden cardiac events. Compared with general anxiety or depression, kinesiophobia is a more specific construct, grounded in the context of physical activity (9, 44). Its distinctive feature lies in the integration of symptom distress, emotional responses, and avoidance behavior, which collectively shape patients' reluctance to exercise. This analysis consequently extends the understanding of psychological barriers to rehabilitation in HF and highlights the unique characteristics of kinesiophobia in this population.

These findings have important clinical implications. Healthcare providers should recognize kinesiophobia as a significant barrier to effective rehabilitation in HF. Routine assessment of fear of movement should be incorporated into patient care. This can be accomplished using validated instruments, most notably the Tampa Scale for Kinesiophobia-Heart (TSK-SV Heart) or its shorter versions, which have been used in cardiac populations (13). Simple screening questions about a patient's specific fears regarding activity can also be effective in identifying those at risk. Once identified, tailored interventions are essential, including patient education to correct cognitive misinterpretations, psychological support to address fear and anxiety, and graded exercise programs to rebuild confidence in physical activity (47, 48). Moreover, family involvement and multidisciplinary collaboration among cardiologists, rehabilitation specialists, nurses, and psychologists are crucial for provide comprehensive support (49, 50). Addressing kinesiophobia could enhance adherence to exercise, improve recovery, and ultimately contribute to better health outcomes and quality of life for HF patients.

Despite growing recognition, gaps remain in the study of kinesiophobia in HF. A standardized definition and population-specific measurement tools are lacking, making it difficult to compare findings across studies. Conceptually, the mechanisms linking antecedents and attributes require deeper investigation, particularly the interplay between psychological factors and disease severity. Notably, the contextual factors influencing kinesiophobia's impact are also under-explored; for example, it is unclear whether its effect on rehabilitation adherence differs between highly supervised outpatient settings and less structured home-based programs. Future research should prioritize longitudinal designs to establish causality, develop and validate HF-specific kinesiophobia scales, and conduct comparative effectiveness trials to tailor interventions for different rehabilitation contexts.

## Limitations

This analysis is subject to several limitations. First, the findings were derived solely on published studies retrieved from selected databases, which may have excluded relevant evidence not indexed in these sources. Second, gray literature was not included, potentially overlooking additional perspectives on kinesiophobia in HF. Third, the analysis was restricted to English-language publications, which may have limited the inclusion of culturally or contextually specific attributes and antecedents reported in non-English literature.

## Conclusion

This concept analysis elucidated the meaning of kinesiophobia in HF patients, identifying surrogate terms, related concepts, four defining attributes, multiple antecedents, and three categories of consequences. Kinesiophobia in HF is a multidimensional construct shaped by the interaction of physical, psychological, and social factors, contributing to physical decline, psychological distress, and impaired disease management. By delineating its characteristics, this study establishes a foundation for future theory development and the creation of targeted interventions. The findings underscore the importance of routine assessment, psychological support, patient education, and multidisciplinary approaches to address fear of movement in HF populations. Future research implications include developing standardized definitions and measurement tools, exploring causal mechanisms, and testing evidence-based interventions to mitigate kinesiophobia and improve health outcomes.
